# Serum Biomarkers of (Anti)Oxidant Status for Epidemiological Studies

**DOI:** 10.3390/ijms161126032

**Published:** 2015-11-16

**Authors:** Eugène Jansen, Tatjana Ruskovska

**Affiliations:** 1 Centre for Health Protection, National Institute for Public Health and the Environment, PO Box 1, 3720 BA Bilthoven, The Netherlands; 2 Faculty of Medical Sciences, Goce Delcev University, 2000 Stip, Macedonia; tatjana.ruskovska@ugd.edu.mk

**Keywords:** biomarkers, nutrition, vitamins, oxidative stress, antioxidant status, redox status, epidemiology

## Abstract

In this review, we disclose a selection of serum/plasma biomarkers of (anti)oxidant status related to nutrition, which can be used for measurements in large-scale epidemiological studies. From personal experience, we have come to the following proposal of a set of biomarkers for nutritional intake, (anti)oxidant status, and redox status. We have selected the individual antioxidant vitamins E and A, and the carotenoids which can be measured in large series by HPLC. In addition, vitamin C was selected, which can be measured by an auto-analyzer or HPLC. As a biomarker for oxidative stress, the ROM assay (reactive oxygen metabolites) was selected; for the redox status, the total thiol assay; and for the total antioxidant status the BAP assay (biological antioxidant potential). All of these biomarkers can be measured in large quantities by an auto-analyzer. Critical points in biomarker validation with respect to blood sampling, storage conditions, and measurements are discussed. With the selected biomarkers, a good set is presented for use in the risk assessment between nutrition and (chronic) diseases in large-scale epidemiological studies. Examples of the successful application of these biomarkers in large international studies are presented.

## 1. Introduction

A healthy and balanced diet, along with regular and rational physical activity and social components of human life, are key elements of the health and welfare of people. There is strong scientific evidence that cardiovascular, metabolic, neurodegenerative, and malignant diseases are closely related to diet [1].

Cardiovascular, neurodegenerative, and malignant diseases have an impaired oxidative status as the common denominator. However, supplementation with antioxidant (pro)vitamins which, in some way, logically results from the oxidative stress hypothesis, is still associated with numerous open questions and controversy. Namely, a meta-analysis of relevant randomized trials found that supplementation with antioxidant (pro)vitamins has no significant effect in primary and secondary prevention, and can even cause an increased mortality, which is the case for β-carotene, vitamin E, and high-doses of vitamin A [2]. However, there is a lot of justified criticism directed to these trials [3,4,5,6]. It is also important to note that analyzing these results one should take into account the possibility of occurrence of so-called “reductive stress”, resulting from the application of very high doses of antioxidant (pro)vitamins that could disrupt the signaling functions of reactive oxygen species.

In contrast, modern science has strong evidence that a rational and balanced diet, rich in a variety of fruits and vegetables, is strongly associated with the reduced risk of chronic non-communicable diseases [7]. Recent studies demonstrated that this kind of diet leads to an improved redox status in healthy individuals, which manifests itself by the values of these biomarkers to determine the level of oxidative stress [8].

Biomarkers of nutrition are often used in epidemiological studies focused on the status of certain nutrients to assess possible deficiencies or overload. The most important advantages for the use of nutrition biomarkers, as compared to the diet assessment methods, are a real assessment of certain intake of nutrients and the resulting physiological concentrations. Although dietary biomarkers generally provide a more proximal measure of dietary intake, other important factors may also contribute to the levels of nutritional biomarkers, such as genetic variability, lifestyle (e.g., smoking), gender, *etc.* Disturbing factors for a good biomarker study are unwanted and unexpected variations due to circadian rhythm, seasonal influences, dietary factors (e.g., postprandial), storage stability, and analytical methods [9].

In this review a number of biomarkers that reflect anti-oxidant nutrition, and oxidative and redox processes, are selected and methods for measurements in large-scale epidemiological studies will be described.

## 2. Biomarker Selection

For most anti-oxidant nutrients, there is one biomarker that reflects the nutritional status. A potential source of undesirable variation in biomarker studies is the existence of daily variations, which can be caused by postprandial or circadian effects. For fat-soluble vitamins, the process of homeostasis will keep the blood levels constant by using tissues as a transient storage compartment. As a result, changes in biomarker concentrations, particularly fat-soluble vitamins, will only be reflected in the long term, which is a good situation for epidemiological studies. Therefore, we focused on the fat-soluble vitamins A and E and carotenoids. In addition, the water-soluble vitamin C may be added to the anti-oxidant vitamins, in order to complete the status.

The result of a good anti-oxidant status can be measured by biomarkers of oxidative stress and redox status. From our experience in large-scale projects, we propose the use of the biomarkers for hydroperoxides and total thiols, which can both be measured in large series on auto-analyzers.

## 3. Biomarkers of Antioxidant Vitamins and Provitamins

### 3.1. Biomarkers of Vitamin A

The preferred biomarker for the status of vitamin A in serum or plasma is retinol. Retinol, however, is not a good biomarker for the determination of small and short-term changes in intakes, for two reasons. Firstly, retinol is a fat-soluble vitamin and, thus, changes in intake will be reflected by this biomarker only after several weeks. Secondly, retinol is under homeostatic control by the liver, which means that deviations from normal concentrations are corrected by the release or storage of hepatic retinol. This makes this biomarker not very suitable for intervention studies, but more suitable for large population studies, because it reflects the long-term status of vitamin A.

**Figure 1:**
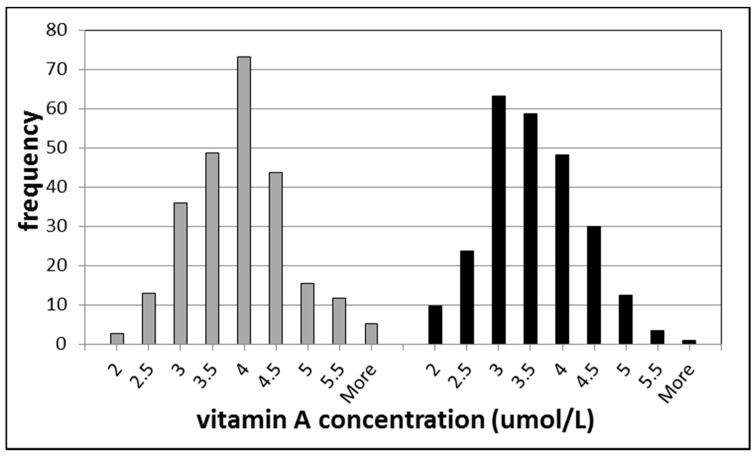
Histograms of vitamin A (retinol) concentrations in healthy individuals. The left part represents the data for men and the right part for women.

The inter-individual variation of vitamin A concentrations in the population is quite large, however. In Figure 1, a histogram of the vitamin A concentrations from a population study of healthy individuals of Amsterdam is shown. A large variation was observed among subjects with inter-individual variation of 23.8% and 23.4% for men and women, respectively [10]. This relatively large variation means that participants in epidemiological studies can be distinguished from each other.

For possible high intakes of vitamin A by supplementation, retinyl esters in serum, or plasma are good biomarkers [11]. These compounds may be detected because of an apparently partial hydrolysis of the supplied esters.

### 3.2. Biomarkers of Vitamin E

**Figure 2:**
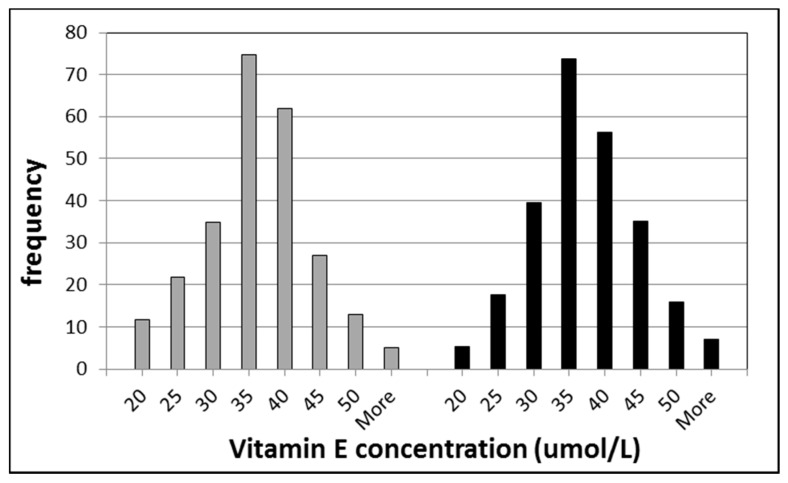
Histograms of vitamin E (the sum of α- and γ-tocopherol) concentrations in healthy individuals. The left part represents the data for men and the right part for women.

The status parameter of vitamin E in serum or plasma is α-tocopherol, although γ-tocopherol is also present to a lower extend. These tocopherols, similarly to retinol, are not very good biomarkers for determining small changes in the nutritional status of vitamin E, as the tocopherols are fat-soluble and under homeostatic control by the liver, as described for vitamin A. In Figure 2, a histogram of the vitamin E concentrations of a population in Amsterdam is shown as also described for vitamin A. Again, it was observed that wide inter-individual differences exist between the individuals; 23.2% and 22.1% for men and women, respectively [10]. Therefore, vitamin E as α-tocopherol can be used preferably for epidemiological studies.

### 3.3. Biomarkers of Carotenoids

The most common dietary carotenoids are α-carotene, β-carotene, β-cryptoxanthin, lutein, zeaxanthin, and lycopene. The biomarkers of carotenoid intake are the parent compounds. These compounds are biomarkers of intake of various fruits and vegetables in the human nutrition [12]. The carotenoids can be divided into carotenes (α-carotene, β-carotene, and lycopene) and xanthophylls (β-cryptoxanthin, lutein, and zeaxanthin) on the basis of their structural moieties. α-Carotene, β-carotene, and β-cryptoxanthin are provitamin A carotenoids, which are converted by the body to retinol (vitamin A).

The carotenoids are also fat-soluble compounds having even greater hydrophobicity than vitamin A and E. Thus, the carotenoids also respond relatively slowly to changes in intake. As a result, these biomarkers are not suitable for transient, but for long-term changes in intake [13]. Many studies of carotenoids have shown these compounds can serve as good biomarkers to indicate a risk of several chronic diseases, such as cardiovascular diseases [14] and cancer [13,15,16].

### 3.4. Biomarker of Vitamin C

The biomarker of vitamin C is the parent compound, which is l-ascorbic acid. Vitamin C is readily water-soluble and will not be stored in tissues. Therefore, vitamin C is a good biomarker for short-term studies, but also useful in epidemiological studies in relation to diseases [13,17,18]. It should be emphasized that vitamin C is relatively unstable in serum or plasma samples. Repeated freeze/thaw cycles will reduce the concentration of vitamin C substantially. In addition, storage at room temperature or even at −20 °C under extended periods of days or weeks, will give erroneous results in the determination of the vitamin C content.

### 3.5. Measurements of Biomarkers of Antioxidant (pro)Vitamins

The preferred biomarker for the status of vitamin A is serum retinol, which may be routinely determined by means of HPLC with UV detection, often together with α- and γ-tocopherol in the same HPLC procedure. In a suitable isocratic reversed phase separation retinol and both α- and γ-tocopherol can be determined by UV-detection or more sensitively by fluorescence.

The carotenoids can also be measured by means of HPLC either separately or in the same run as vitamins A and E. Carotenoids can be measured with detection at a visible wavelength of 450 nm [13]. Up to seven different carotenoids can be detected simultaneously being α- and β-carotene, zeaxanthin, lutein, canthaxanthin, β-cryptoxanthin, and lycopene [13,19]. In addition, three peaks of lycopene can be detected representing an all-trans isomer and a number of *cis*-isomers of lycopene [13].

**Figure 3:**
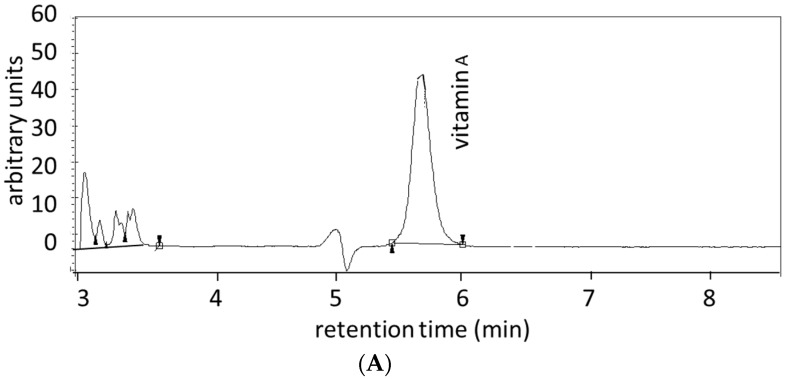

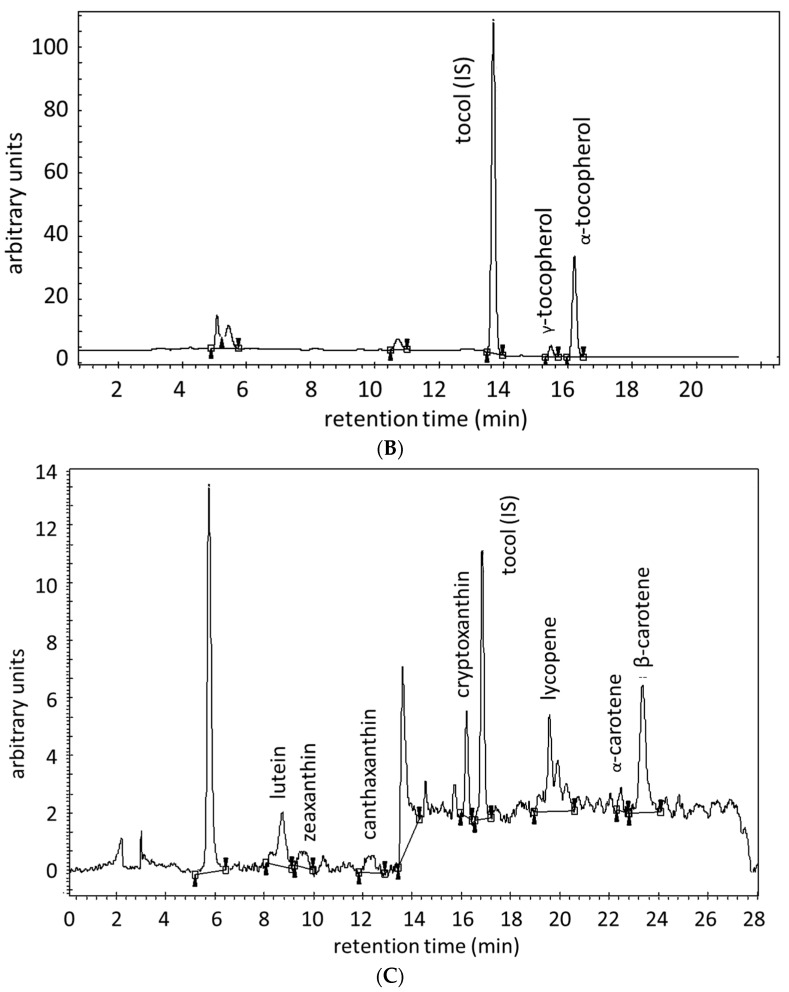
Chromatogram of the separation and detection of vitamin A (**A**); α- and γ- tocopherol (**B**); and seven carotenoids (**C**) in the same HPLC procedure. These HPLC chromatograms are from the study described in reference 13 [13].

In the literature, there are numerous reports on the combined analysis of retinol, tocopherols, and carotenoids in serum or plasma [12,13,14,19,20]. Shortly, after addition of an internal standard, a solvent extraction is carried out. This extract can be applied onto a reversed phase column and eluted with a stepwise gradient. An example is given in Figure 3 wherein a typical chromatogram of a serum extract is shown with UV detection of vitamin A (at 325 nm), vitamin E by fluorescence (excitation at 225 nm and emission at 325 nm), and visible wavelength detection of carotenoids (at 450 nm).

**Figure 4:**
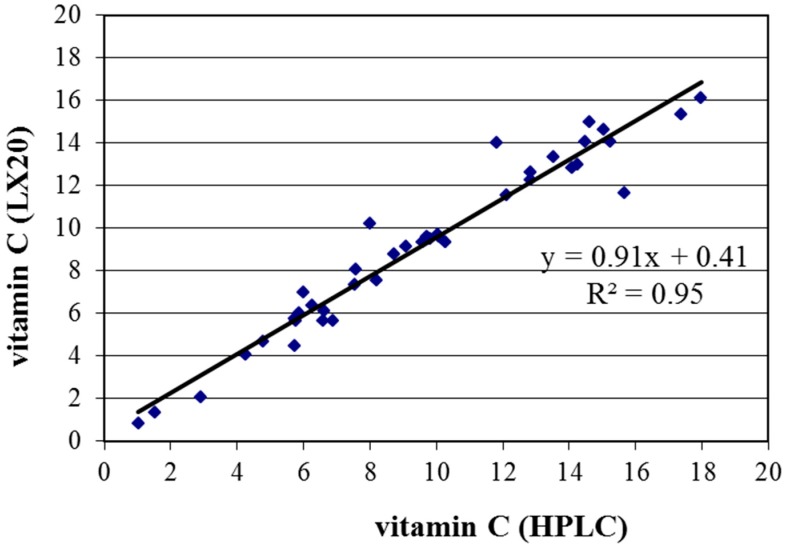
Comparison of the vitamin C determination with HPLC and with a colorimetric method as measured on an auto-analyzer (LX20, Beckman-Coulter, Woerden, The Netherlands).

Vitamin C exists as two redox forms being ascorbic acid (reduced form) and dehydroascorbic acid (oxidized form), which are in equilibrium. In the analysis of vitamin C, the equilibrium between the two forms need to be fully shifted to one form, which is then determined by HPLC [21] or a colorimetric assay [22]. In both cases, a deproteinization step is required, that this assay does not readily lend themselves to automatic analyzers but only after a pre-analytical step. Both of these methods can be applied with good results. We conducted a small study comparing the HPLC and colorimetric method with 40 serum samples of healthy volunteers. The result is shown in Figure 4. A very good correlation was observed between the two methods with *R*^2^ = 0.95.

## 4. Biomarkers of Total Antioxidant Defense

Methods for the quantification of the non-enzymatic plasma antioxidant activity can be divided into two groups: (a) methods for measurement of concentrations of individual antioxidant (pro)vitamins, covered in previous section; and (b) methods for the determination of the total non-enzymatic plasma antioxidant capacity.

In terms of their specificity, the advantage of the methods for measuring the concentrations of individual nutrients with antioxidant activity is beyond dispute. On the other hand, the possibility for screening the “total” plasma antioxidant capacity with fast, simple, and inexpensive methods is particularly attractive and practical. Therefore, in clinical and epidemiological studies of chronic diseases associated with oxidative stress in general, as well as in those that are specifically related to oxidative stress and diet, the use of total antioxidant assays is very common.

There is a great variety of methods for determining the total antioxidant capacity, which can also be used for samples of blood plasma or serum [23]. The available methods are based on different principles, and the relative contribution of individual antioxidants to the total antioxidant activity is also variable. This can cause large differences in the correlation between these methods [24].

Some of the most reliable and widely-used methods for the determination of the total non-enzymatic antioxidant capacity of blood plasma or serum, which are suitable for application in large-scale clinical or epidemiological studies, are as follows: (a) methods in which 2,2′-azino-di-(3-ethylbenzthiazoline sulphonate) radical cation (ABTS*^+^, * is an unpaired electron) is used as an indicator of antioxidant activity, such as TAS assay (RelAssay, Gaziantep, Turkey); (b) the method known as Ferric Reducing Ability of Plasma (FRAP); and (c) the test known as Biological Antioxidant Potential (BAP, Diacron, Grosseto, Italy). In addition to these, there are many other methods for the quantification of the total non-enzymatic plasma antioxidant capacity, such as an enzymatic peroxidase-based method ImAnOx (Immundiagnostik AntiOxidant), the hypochlorous-based assay OxyAdsorbent (Diacron, Grosseto, Italy), and Oxygen Radical Absorbance Capacity (ORAC).

For their use in human studies relating to nutrition and disease, a first approach must be the selection of antioxidant assays which are not affected by non-nutritional components in serum. The BAP assay has a relatively low correlation with uric acid and therefore can be a good choice [24]. In addition, other components can be measured in the same analysis procedure to assist the results obtained by the BAP assay. These components are uric acid, albumin, or total serum proteins and, to a lesser extent, bilirubin and creatinine. Several other assays are rather laborious for use in epidemiological studies, such as ORAC and ImAnOx.

The measurement of the total antioxidant status remains an important and easy to determine biomarker. In combination with other biomarkers and clinical data, it can be used for large-scale or intervention studies. Moreover, the non-enzymatic plasma antioxidant capacity, of course, includes the concentration of total thiols as well, but this assay will be considered in section 5.

## 5. Biomarkers of Oxidant Status

Biomarkers of oxidant status can be used for assessment of the oxidative/antioxidative balance with regard to nutrition or diseases. There are many assays that represent oxidative stress processes such as: malondialdehyde (MDA), 8-hydroxy-2′-deoxyguanosine (8-OHdG), reactive oxygen metabolites (ROM), total oxidant status (TOS), hydroperoxides enzymatic (PerOx), and oxidized proteins (Ox-Prot). In this review, we will restrict to assays that can be performed in serum or plasma samples in relatively large series of epidemiological studies and can be included in a multi-component analysis.

It should be emphasized that production of free radicals increased significantly during acute infection, which is in function of defense of the organism from pathogens [25]. The use of CRP as a biomarker for transient infections can be useful to exclude samples from individuals suffering from high oxidative stress as a result of non-chronic inflammation.

### 5.1. Biomarkers of Lipid Peroxidation

MDA and 4-hydroxynonenal are highly reactive aldehydes, which are end-products of lipid peroxidation. For MDA a number of assays are commercially available, which are based on colorimetric or fluorescence detection. These assays are not very specific because of the simple pretreatment. More specific assays are based on a chromatographic pre-purification step by HPLC. Several companies sell these HPLC assays that use a completely different sequence of pretreatment and derivatization procedures. In our hands, these assays gave results which do not correlate well with each other. Taken into account the rather laborious assay procedure and results obtained in our laboratory and in the literature, we do not recommend these assays for epidemiological studies.

### 5.2. Biomarkers Based on Hydroperoxide Detection

In recent years there has been an increase in the number of research papers that correlate general biomarkers of oxidative stress with chronic diseases. Most of these assays, such as ROM assay (Reactive Oxygen Metabolites) from Diacron, Grosseto, Italy and TOS (Total Oxidant Status) from RelAssay, Gaziantep, Turkey, are based on the iron-mediated reactions of apparently-stable products (hydroperoxides) of the lipid peroxidation process. Various commercial assays use various dye molecules, which are closely related. In contrast, the PerOx assay (test kit from Immundiagnostik, Bensheim, Germany) is based on an enzymatic reaction using peroxidase for the oxidation of the dye molecules.

The advantage of non-enzymatic, iron-based oxidation assays is the possibility for automation. This can be performed on automated clinical analyzers or in microtiter plate formats, which allows to process large quantities of samples.

Although these assays are based on simple chemistry, which can be set up in all laboratory settings, the main advantages of using commercial kits are a good quality control and assay stability. This seems to be the main difficulty in obtaining reliable results in large-scale studies which require assay stability for a longer period of time. The ROM assay, which is an iron-catalyzed colorimetric reaction with *N*,*N*-diethyl-*p*-phenylenediamine as substrate, can be applied to various programmable clinical auto-analyzers. In addition, the stability of the hydroperoxides in serum stored for many years seems to be good. We tested the stability of the ROM assay for one year of storage of serum samples at −20 and −80 °C with very good results [26]. Studies are ongoing to evaluate the stability of some of these biomarkers to eight years.

The positive correlation with colorectal cancer in samples that were stored for about 25 years in liquid nitrogen, give us a reliable feeling that this biomarker can be used on these rather old samples [27]. In addition, the short-term stability during several days of assay operation is also very good [28]. Moreover, short-term factors such as circadian rhythm or a postprandial effect does not seem to affect this biomarker [29].

The ROM assay has been used for several years and has proven its value in large-scale studies. In a large European project (CHANCES), oxidative stress was recently introduced as a biomarker [30]. The ROM assay has been correlated with cardiovascular disease [31,32], cancer [27,33,34,35], all-cause mortality [36], and aging [37].

Other assays that represent oxidative stress processes are those measuring oxidation products of DNA. These assays have also proven their value in the research of risk for cancer. Due to the complexity and required high sensitivity, such assays are not suitable for routine analysis. The present enzyme immunoassays do not appear sensitive enough for measurements in serum or plasma samples without a pre-concentration step.

### 5.3. Biomarkers of Redox Status

Oxidation processes will also occur in proteins and at the DNA level. One of the most promising biomarkers of protein oxidation is the determination of total thiol levels in serum or plasma [38]. The number of free thiol groups as cysteine residues in proteins is a measure for a good redox status. A reduced number of thiol groups is an unfavorable situation. Therefore, the assay for total thiol groups can be used both as an oxidation assay and as an antioxidant status assay. Total thiols can be measured with Ellman’s reagent, which is a solution of 5,5′-dithio-bis-(2-nitrobenzoic acid), also known as DTNB. In this method, the DTNB reacts with free sulfhydryl groups producing a yellow colored product [39,40]. The total thiol assay can be obtained from a number of companies. Particularly, kits from RelAssay (TTL assay) and Diacron (SHp assay) can be used for automated systems as clinical auto-analyzers.

The total thiol assay has been used successfully in a large European project (CHANCES) [30]. The total thiol assay was correlated with all-cause mortality [36] and aging [37].

Other biomarkers of redox processes can be found in the glutathione pathway, with glutathione (GSH) and oxidized glutathione (GSSG) as the most important biomarkers. Often the ratio of the two components has been measured. In serum or plasma, however, the concentration of GSH is much lower than in the erythrocytes. As a result, the detection of GSH in serum or plasma often reflects leakage of GSH from the erythrocytes and, therefore, serum or plasma are not suitable for GSH measurements. To assess the redox status, total thiols are to be the preferred biomarker.

## 6. Conclusions

Accurate and reliable biomarkers that reflect nutrient intake, status, and effect are of great importance [41,42,43]. However, there is still no broad consensus in relation to their use and application. In this review article we focused on nutritional antioxidant biomarkers and biomarkers that reflect the human body’s oxidative stress and redox status that can be measured in relatively large number of samples using auto-analyzers and HPLC methods. Moreover, this review focuses on biomarkers for long-term effects that will be used in epidemiological cohort studies.

With regards to plasma concentrations of vitamin C and carotenoids, there is consensus that they are good biomarkers for providing general information about the intake of fruit and vegetables [42]. It is pointed out that vitamin C is sensitive to pre-analytical conditions and also to short-term effects.

For the fat-soluble antioxidant vitamins A and E, and the carotenoids, the serum concentrations of these biomarkers need some weeks to reach an equilibrium between fat and blood levels. Consequently, caution is advised when they are used in short-term studies. However, in long-term studies, these biomarkers are suitable for risk analysis in large epidemiological cohort studies on chronic diseases.

For the oxidant status, ROM is a good biomarker for oxidative stress, because ROM has proven its value in many studies. Furthermore, the total thiols give an additional indication about the (anti)oxidant and redox status in serum or plasma.

Measurement of total antioxidant capacity in plasma or serum is a simple and inexpensive method to assess changes in the anti-oxidant status. This biomarker was measured in numerous clinical studies related to nutrition [44], in combination with other biomarkers of oxidative stress and clinical data [45,46,47]. For the antioxidant status, BAP is a good biomarker because it is easy to measure, as the TAS assay, but the BAP assay has a much lower contribution from uric acid in comparison with similar antioxidant assays.

The characteristics of the biomarkers that are selected for epidemiological studies in this review have been summarized in Table 1, including the expected normal and unhealthy concentration ranges, where these are known from literature (see references [48,49,50]) or given by the commercial companies of the assays. For carotenoids, vitamin A, and vitamin E, the normal ranges are dependent on the analytical methods used and also on the different populations or countries [51].

**Table 1:** Characteristics of the biomarkers selected for epidemiological studies.

Biomarker	Time Range	Normal Range	Unhealthy Range	Method of Analysis	Multi-Component	Samples/Day
Vitamin A	weeks-months	1.8–7.0 µmol/L [48]	no data	HPLC	yes	80
Vitamin E	weeks-months	14–40 µmol/L [49]	<21 µmol/L [50]	HPLC	yes	80
Carotenoids	weeks-months	50–650 µg/L [49]	no data	HPLC	yes	80
Vitamin C	hours-days	4.1–20.0 mg/L [49]	2.0–4.1 mg/L [49]	HPLC or Auto-Analyzer	no	100
BAP	days-months	>2200 µmol/L	<2000 µmol/L	Auto-Analyzer	yes	240
ROM	days-months	250–300 Carr·U	>320 Carr·U	Auto-Analyzer	yes	240
TTL	days-months	no data	no data	Auto-Analyzer	yes	240
SHp	days-months	450–650 µmol/L	<450 µmol/L	Auto-Analyzer	yes	240

**Figure 5:**
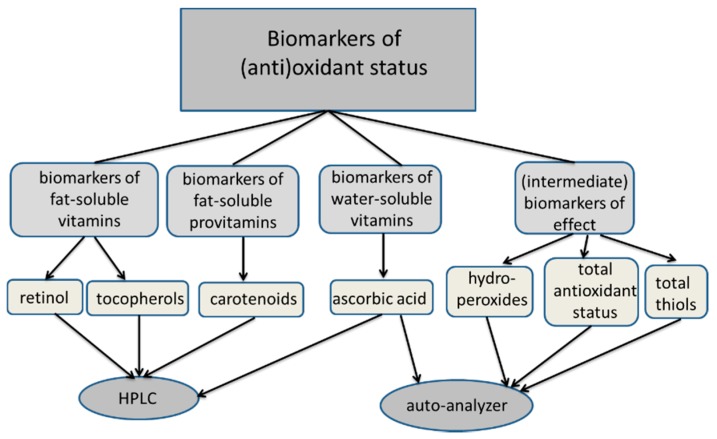
Proposed scheme for the measurement of biomarkers of (anti)oxidant status related to nutrition and chronic diseases in large-scale studies.

Finally, we come to the following proposal of a set of biomarkers to determine the (anti)oxidant status in serum as a reflection of nutrition in large number of samples in epidemiological studies (see Figure 5). With the combination of vitamin A, vitamin E, carotenoids, vitamin C, ROM, BAP, and total thiols, an overall image is obtained concerning the intake by nutrition and effect of antioxidants on disease risk.
